# Sleeping Together

**Published:** 1877-07

**Authors:** 


### SLEEPING TOGETHER.

If a man were to see a quarter of an inch of worm put in his cup of coffee, he could not drink it, because he knows that the whole cup would be impregnated. If a very small amount of some virulent poison be introduced into a glass of water, the drinking of it might not produce instant death, but that would not prove that it was not hurtful, only that there was not enough of it to cause a destructive result immediately.

We sicken at the thought of taking the breath of another the moment it leaves the mouth, but that breath mingles with the air about the bed, in which two persons lay; and it is re-breathed, but not the less offensive is it in reality, on account of the dilution, except that it is not taken in its concentrated form, but each breath makes it more concentrated. One sleeper corrupts the atmosphere of the room by his own breathing, but when two persons are breathing at the same time, twelve or fourteen times in each minute, in each minute extracting all the nutriment from a gallon of air, the deterioration must be rapid indeed, especially in a small and close room. A bird cannot live without a large supply of pure air. A canary bird hung up in a curtained bedstead where two persons slept, died before the morning.

Many infants are found dead in bed, and it is attributed to having been over laid by the parents, but the idea that any person could lay still for a moment on a baby or any thing else of the same size, is absurd. Death was caused by the want of pure air.

Besides, emanations ærial and more or less solid, are thrown out from every person, thrown out by the processes of nature, because no longer fit for life purposes, because they are dead and corrupt, but if breathed into another living body, it is just as abhorrent as if we took into our mouths the matter of a sore or any other excretion.

The most destructive typhoid and putrid fevers are known to arise directly from a number of persons living in the same small room.

Those who can afford it, should therefore arrange to have each member of the family sleep in a separate bed. If persons must sleep in the same bed, they should be about the same age, and in good health. If the health be much unequal, both will suffer, but the healthier one the most, the invalid suffering for want of an entirely pure air.

So many cases are mentioned in standard medical works, where healthy, robust infants and larger children have dwindled away, and died in a few months from sleeping with grand parents, or other old persons, that it is useless to cite special instances in proof.

